# *Prunella vulgaris* Compound Improves Sleep Architecture and Attenuates Hippocampal Injury in Insomnia Mice

**DOI:** 10.3390/life16081268

**Published:** 2026-07-31

**Authors:** Kai Wang, Haojia Zhang, Chang Zhou, Zijin Sun, Wei Shao, Kunjing Liu, Wenyuan Ma, Chunyu Wang, Xueqian Wang, Qingguo Wang, Yanyan Jiang, Fafeng Cheng

**Affiliations:** 1 School of Traditional Chinese Medicine, Beijing University of Chinese Medicine, Beijing 102446, China; 20250941032@bucm.edu.cn (K.W.);; 2 School of Chinese Materia Medica, Beijing University of Chinese Medicine, Beijing 102446, China

**Keywords:** *Prunella vulgaris* compound, insomnia, sleep architecture, REM, neurotransmitters, NLRP3 inflammasome, pyroptosis, hippocampus

## Abstract

Insomnia is associated with disrupted sleep architecture, hippocampal pathological alterations, neurotransmitter imbalance, and neuroinflammatory responses. This study evaluated the effects of the Prunella vulgaris compound (PVC) on sleep architecture and hippocampal alterations in p-chlorophenylalanine (PCPA)-induced insomnia model mice and examined their association with NLRP3-related pyroptotic signaling. PVC was characterized using UPLC-Q-Exactive Orbitrap MS and targeted HPLC quantification. PCPA-induced insomnia models were established in ICR and C57BL/6N mice for pharmacodynamic and molecular assessments and EEG/EMG recording, respectively. Sleep outcomes were assessed using the pentobarbital-induced sleep test and EEG/EMG; hippocampal histomorphology, neurotransmitters, and inflammasome- and pyroptosis-related markers were evaluated using histological, biochemical, RT-qPCR, and Western blot analyses. UPLC-MS tentatively characterized 198 constituents, and salviaflaside, rosmarinic acid, and linarin were quantified by HPLC. PVC shortened sleep latency, prolonged sleep duration, reduced wakefulness, increased NREM and REM sleep, and improved sleep continuity. It also attenuated hippocampal histomorphological alterations; modulated 5-HT, GABA, glutamate, and dopamine levels; and reduced NLRP3 inflammasome- and pyroptosis-related markers together with IL-1β and IL-18. PVC improved insomnia-like phenotypes and attenuated hippocampal pathological alterations in PCPA-induced insomnia model mice. These effects were associated with improved sleep architecture and neurotransmitter homeostasis and with reduced NLRP3/caspase-1/GSDMD-related signaling.

## 1. Introduction

Sleep is a fundamental physiological process essential for maintaining systemic homeostasis and mental health and is jointly regulated by the nervous, endocrine, and immune systems [1,2]. Insomnia is one of the most prevalent sleep disorders and is characterized by persistent difficulty in falling asleep, maintaining sleep, or waking up prematurely despite adequate opportunity for sleep, often accompanied by daytime impairment [3]. The global prevalence of insomnia is estimated to range from 10% to 30% and may reach 38.2% to 57% in certain regions of Asia [4,5]. In recent years, accelerated urbanization, intensified occupational competition, and the rapid advancement of information technology have rendered high mental stress increasingly prevalent. Irregular sleep schedules, prolonged overtime work, and excessive use of electronic devices before bedtime further disrupt circadian rhythms, thereby exacerbating difficulty in sleep initiation [6]. Under the combined effects of these factors, the prevalence of insomnia has continued to rise, and growing evidence indicates that chronic insomnia is associated with daytime dysfunction and cognitive or affective disturbances [7].

Normal sleep consists of the cyclical alternation between non-rapid eye movement (NREM) sleep and rapid eye movement (REM) sleep. NREM sleep, particularly slow-wave sleep, is primarily involved in physical restoration, whereas REM sleep is closely associated with memory consolidation and emotional regulation [8]. The hippocampus is a key structure involved in the regulation of the sleep–wake cycle and is closely linked to both NREM and REM sleep [9,10]. Insomnia not only reduces total sleep time, but also disrupts the balance between NREM and REM sleep, thereby affecting hippocampus-related neural processes [11]. Previous studies have further indicated that chronic sleep disturbance may impair hippocampal synaptic plasticity, promote neuroinflammatory responses, and increase vulnerability to cognitive and emotional dysfunction [12,13]. These findings suggest that hippocampal dysfunction may serve as an important link between disrupted sleep architecture and insomnia-associated neurological consequences.

From a mechanistic perspective, insomnia may activate the hippocampal NOD-like receptor family pyrin domain-containing 3 (NLRP3) inflammasome, thereby triggering caspase-1-dependent gasdermin D (GSDMD) cleavage and inducing pyroptosis [14]. The NLRP3 inflammasome has been increasingly recognized as a central mediator of neuroinflammation in sleep disturbance-related neural injury. Once activated, this pathway can amplify inflammatory signaling and contribute to neuronal dysfunction through the release of inflammatory cytokines and the induction of pyroptotic cell death. This process may contribute to hippocampal pathological alterations and neurotransmitter imbalance, thereby affecting sleep homeostasis and sleep architecture [15].

The NLRP3 inflammasome is a canonical inflammasome complex that is activated in response to cellular damage or inflammatory stimuli. Upon activation, it promotes the release of proinflammatory cytokines such as interleukin-1β (IL-1β) and interleukin-18 (IL-18) and induces pyroptosis through caspase-1-mediated cleavage of GSDMD [16]. Accordingly, NLRP3 and ASC were selected as markers of inflammasome assembly, caspase-1 and GSDMD as markers of pyroptotic execution, and IL-1β and IL-18 as downstream inflammatory mediators. As a recently characterized mode of programmed cell death, pyroptosis has emerged as a focal point in research on immune responses and neurodegenerative disorders [17]. Within the nervous system, NLRP3-mediated pyroptosis contributes to neuronal injury and cognitive dysfunction [18]. As a key brain region involved in learning and memory, the hippocampus is closely associated with cognitive impairment when its function is compromised. Therefore, preserving hippocampal function and inhibiting pyroptosis-related neuroinflammatory responses may represent an effective strategy for alleviating cognitive dysfunction [19].

Currently, pharmacological treatment of insomnia primarily depends on benzodiazepines, non-benzodiazepine agents, and melatonin-based medications, which mainly exert their effects by enhancing GABAergic or melatonin receptor signaling [20]. However, prolonged use of these medications is frequently associated with tolerance, dependence, and withdrawal-related rebound insomnia and may further compromise physiological sleep architecture, manifesting as excessive prolongation of NREM sleep, significant shortening of REM sleep, and increased sleep fragmentation [21]. These adverse effects limit their long-term use in the treatment of chronic insomnia, underscoring the urgent need to develop safe and effective alternative therapies capable of restoring natural sleep rhythms [22]. In this context, multi-component herbal formulations may provide a complementary strategy because they can simultaneously target sleep regulation, neurotransmitter balance, and neuroinflammatory pathways.

In our previous studies, *Prunella vulgaris* components demonstrated promising therapeutic efficacy against insomnia. In contrast to conventional Western medicines, traditional herbal formulations are distinguished by their multi-component and multi-target characteristics, allowing them to produce synergistic effects in insomnia treatment [23]. Owing to the synergistic effects of their herbal constituents, these formulations may enhance therapeutic efficacy while reducing adverse effects [24]. The Prunella vulgaris compound (PVC) consists of five medicinal herbs, which collectively exert synergistic effects in alleviating insomnia. Previous studies have reported that Prunella vulgaris and its active constituents exhibit anti-insomnia and sedative-hypnotic-like effects, which provided a pharmacological basis for selecting Prunella vulgaris as the core herb in the present formula [25]. However, research on the protective effects of the PVC against insomnia-induced neuronal injury and learning and memory deficits remains scarce, and the underlying mechanisms require further elucidation.

Therefore, the present study aimed to systematically investigate the effects of the PVC on sleep architecture, neurotransmitter alterations, and hippocampal pathological changes in insomnia model mice. In addition, we sought to examine whether these effects were associated with NLRP3 inflammasome- and pyroptosis-related molecular signaling. This study was expected to provide experimental evidence for the potential use of PVC in improving insomnia-like phenotypes and related hippocampal pathological alterations.

## 2. Materials and Methods

### 2.1. Herbal Materials and Preparation of the PVC

All herbal materials used for the preparation of PVC were authenticated by Professor Yanyan Jiang from the School of Chinese Materia Medica, Beijing University of Chinese Medicine, Beijing, China. PVC is a multi-herb formulation consisting of Prunella vulgaris, Acorus tatarinowii, Platycladus orientalis seeds, Cirsium setosum, and Bambusa tuldoides. Prunella vulgaris was selected as the core herb based on our previous anti-insomnia screening work, and the remaining herbal components were incorporated according to traditional Chinese medicine formulation principles. To improve reproducibility, PVC was prepared using a fixed extraction and processing protocol throughout the study.

Briefly, Acorus tatarinowii and Platycladus orientalis seeds were first subjected to steam distillation to extract volatile oils. The aqueous distillate, herbal residues, and volatile oil fraction were collected separately. Prunella vulgaris, Cirsium setosum, and Bambusa tuldoides were then mixed with the residues obtained after volatile oil extraction and extracted twice with an 18-fold volume of deionized water for 1.5 h each time after soaking for 1 h. The aqueous distillate and the two filtrates were combined and concentrated to a relative density of 1.10–1.15. Ethanol was added to the concentrated extract until the final ethanol concentration reached 70%. After standing and centrifugation, the supernatant was collected, and the solvent was recovered. The residue was dried under reduced pressure to obtain the dry extract. The volatile oils were encapsulated with β-cyclodextrin and then uniformly mixed with the dry extract. Dextrin was added as an excipient, followed by granulation, drying, and packaging to obtain the final PVC granules.

### 2.2. UPLC-Q-Exactive Orbitrap MS Analysis of the Chemical Profile and Targeted HPLC Quantification of PVC

The chemical profile of PVC was characterized using ultra-performance liquid chromatography coupled with Q-Exactive Focus Orbitrap mass spectrometry (Thermo Fisher Scientific, Bremen, Germany) (UPLC-Q-Exactive Orbitrap MS). Briefly, 5 g of PVC granules were finely powdered, accurately weighed, and placed into a stoppered conical flask. Then, 50 mL of 50% methanol was accurately added, and the total weight was recorded. The sample was ultrasonicated for 60 min and then cooled to room temperature. The weight loss was compensated with 50% methanol, and the extract was mixed thoroughly and allowed to stand. The supernatant was filtered through a 0.22 μm membrane before UPLC-MS analysis.

Chromatographic separation was performed on a Waters Acquity UPLC BEH C18 column (2.1 × 150 mm, 1.7 μm). The mobile phase consisted of methanol (A) and 0.1% formic acid in water (B). The gradient elution program was as follows: 0–3 min, 5% A; 3–43 min, 5–85% A; 43–45 min, 85% A; 45–45.5 min, 85–5% A; and 45.5–50 min, 5% A. The flow rate was 0.3 mL/min, the column temperature was maintained at 30 °C, and the injection volume was 10 μL.

Mass spectrometric detection was performed using the Q-Exactive Focus mass spectrometer equipped with an electrospray ionization (ESI) source. Data were acquired in both positive and negative ion modes using Xcalibur 2.0 software (Thermo Fisher Scientific, Waltham, MA, USA). The optimized MS parameters were as follows: spray voltage, 3 kV; capillary temperature, 350 °C; sheath gas flow rate, 30 arb; auxiliary gas flow rate, 10 arb; normalized collision energy, 35%; activation energy, 0.25 q; and activation time, 30 ms. Full-scan high-resolution MS data were acquired over an *m*/*z* range of 100–1500 with a resolution of 30,000. MS/MS data were obtained using a data-dependent acquisition mode (Full MS/dd-MS^2^) to acquire precursor ion and fragment ion information. Representative chemical constituents were tentatively identified based on retention behavior, accurate mass, and MS/MS fragment ions by comparison with reference standards, databases, or previously published data.

Based on our previous studies identifying salviaflaside and rosmarinic acid as potential sleep-improvement-related constituents of Prunella vulgaris, these two compounds were selected as quantitative chemical markers. Linarin was additionally included as a representative marker attributable to Cirsium setosum in the PVC formulation. Reference standards of salviaflaside were obtained from Sahn Chemical Technology Co., Ltd. (Shanghai, China), whereas rosmarinic acid and linarin were obtained from Shanghai Yuanye Bio-Technology Co., Ltd. (Shanghai, China).

For targeted HPLC quantification, PVC granules were finely powdered, and approximately 100 mg of the powder was accurately weighed and transferred into a 10 mL volumetric flask. The sample was dissolved by ultrasonication in 50% methanol, diluted to volume with 50% methanol, thoroughly mixed, and filtered through a 0.45 μm membrane. Chromatographic separation was performed using a Waters e2695 HPLC system (Waters Corporation, Milford, MA, USA) equipped with an Agilent Extend C18 column (Agilent Technologies, Santa Clara, CA, USA) (4.6 × 250 mm) maintained at 30 °C. The mobile phase consisted of methanol (A) and 0.1% formic acid in water (B), with the following gradient: 0–5 min, 27–29% A; 5–10 min, 29–32% A; 10–30 min, 32% A; 30–35 min, 32–38% A; 35–40 min, 38–48% A; 40–45 min, 48% A; 45–55 min, 48–62% A; and 55–60 min, 62% A. The flow rate was 1.0 mL/min, and the injection volume was 20 μL. Salviaflaside, rosmarinic acid, and linarin were quantified using external-standard calibration at 320, 330, and 334 nm, respectively. The quantitative results are presented in Supplementary Table S2.

### 2.3. Drugs and Reagents

DL-4-chlorophenylalanine (PCPA) was purchased from Aladdin Reagents (Shanghai, China). Diazepam (DZP), used as the positive control drug, was obtained from Beijing Yimin Pharmaceutical Co., Ltd. (Beijing, China). Sodium pentobarbital was purchased from Beijing Chemical Reagent Company (Beijing, China). Normal saline was obtained from Guangxi Yuyuan Pharmaceutical Co., Ltd. (Qinzhou, China). Sodium bicarbonate was purchased from Beijing InnoChem Science & Technology Co., Ltd. (Beijing, China), and Tween 80 was obtained from Beijing Ouhe Technology Co., Ltd. (Beijing, China).

### 2.4. Animals

A total of 102 healthy specific pathogen-free (SPF) male ICR mice and 24 male C57BL/6N mice, aged 8 weeks and weighing 20–25 g, were used in this study. The ICR mice were used for the sodium pentobarbital-induced sleep test and molecular biological experiments, whereas the C57BL/6N mice were used for electroencephalographic monitoring. This experimental design was adopted because ICR mice are widely used in behavioral pharmacology studies, while C57BL/6N mice are more suitable for electrophysiological recording and sleep-stage analysis following electrode implantation. The two strains were used for different experimental endpoints rather than for direct strain-to-strain comparison; therefore, sleep-related outcomes were interpreted within each corresponding experimental system. Experimental grouping and randomization were performed using a random number table. To minimize potential confounding effects, drug administration, behavioral testing, sample collection, and EEG/EMG recordings were performed within the same time windows whenever applicable. Group allocation was known to the investigators responsible for drug administration, whereas the investigators responsible for EEG/EMG sleep-stage scoring and data extraction were blinded to treatment allocation. PVCL, PVCM, and PVCH refer to the low-, medium-, and high-dose PVC groups, respectively, administered at 0.43, 0.86, and 1.72 g/kg. The medium dose of 0.86 g/kg was determined with reference to the adult daily prescription dose of 55 g of crude herbs for a 70 kg adult. The low and high doses were set at 0.5- and 2-fold the medium dose, respectively. In the pharmacodynamic experiment without model induction, ICR mice were randomly assigned to five groups (*n* = 6 per group): control, diazepam, PVCL, PVCM, and PVCH. In the pharmacodynamic experiment with model induction, ICR mice were randomly assigned to six groups (*n* = 12 per group): control, model, diazepam, PVCL, PVCM, and PVCH. In the electroencephalography (EEG) experiment, C57BL/6N mice were randomly assigned to four groups (*n* = 6 per group): control, model, diazepam, and PVC. Based on the pharmacodynamic results, PVCM was selected as the PVC intervention group for EEG/EMG analysis. The primary outcomes were sleep latency, sleep duration, and EEG/EMG-based sleep-stage distribution. Secondary outcomes included body weight change, hippocampal histomorphology, neurotransmitter levels, inflammatory cytokines, and NLRP3 inflammasome-related markers. Body weight was recorded before and after treatment. Animals were excluded if they showed failed EEG/EMG electrode implantation, unstable EEG/EMG signals, or severe postoperative complications or reached humane endpoints. All animals were supplied by Beijing SPF Biotechnology Co., Ltd. and housed in the animal facility of Beijing University of Chinese Medicine under a 12 h light/dark cycle (6:00–18:00) at a temperature of 23 ± 1 °C and a relative humidity of 30–40%, with free access to food and water. All animal procedures were approved by the Animal Care and Use Committee of Beijing University of Chinese Medicine. The ethical approval number for this study was BUCM-2024111505-4188.

### 2.5. Sodium Pentobarbital-Induced Sleep Test

The sodium pentobarbital-induced sleep test (PIST) was used to evaluate sleep in mice, and all experiments were conducted between 9:00 and 17:00. Sleep status was determined by observing the righting reflex, as previously described [26]. When a mouse remained in the supine position for more than 1 min without being able to right itself, the righting reflex was considered lost, indicating sleep onset; restoration of the normal posture was regarded as awakening. Sodium pentobarbital, as a hypnotic agent, can rapidly induce sleep. When it acts synergistically with the tested drug, sleep latency is typically shortened and total sleep time is prolonged. Sleep latency was defined as the time from sodium pentobarbital injection to the loss of the righting reflex, whereas sleep duration was defined as the time from loss to recovery of the righting reflex. According to our preliminary study, the suprathreshold dose of sodium pentobarbital in ICR mice was 50 mg/kg, which is consistent with that reported in recent studies [27].

### 2.6. Establishment of a PCPA-Induced Insomnia Mouse Model

The PCPA-induced insomnia model was established according to a previously reported method. Briefly, mice in the model group were intraperitoneally injected with a PCPA (a tryptophan hydroxylase inhibitor that depletes 5-hydroxytryptamine and disrupts the sleep–wake cycle) suspension at 400 mg/kg once daily for 3 consecutive days, whereas control mice received an equal volume of the vehicle. To prepare the PCPA suspension, sodium bicarbonate powder was dissolved in 0.9% normal saline to obtain a weakly alkaline solution with a pH of approximately 8, as confirmed using pH test strips. PCPA powder was then dissolved in the alkaline solution at a dose of 400 mg/kg, and the mixture was vigorously shaken to ensure complete dissolution. Tween 80 (5%) was added to facilitate dissolution. The PCPA suspension was then subjected to ultrasonic treatment for 30 min before use.

### 2.7. Electrode Implantation for EEG Monitoring

Surgery was performed in the morning. Before surgery, C57BL/6N mice were anesthetized with 0.3% sodium pentobarbital solution (0.15 mL/10 g body weight) and fixed in a prone position on a stereotaxic apparatus. After shaving, a midline scalp incision was made, and the muscle and periosteum were gently separated to expose the skull. Burr holes were drilled at the following coordinates: 2–3 mm anterior to the bregma and 2–3 mm lateral to the left side of the sagittal suture for the positive cortical electrode; 2–3 mm posterior to the bregma and 2–3 mm lateral to the right side of the sagittal suture for the negative cortical electrode; and 2–3 mm posterior to the bregma and 2–3 mm lateral to the left side of the sagittal suture for the ground electrode. Three 1 mm stainless steel screws with conductive silver wires were implanted into the skull so that they contacted, but did not penetrate, the dura mater, and were fixed with a thin layer of glass ionomer cement. Two conductive silver wires, approximately 3 cm in length and 0.2 mm in diameter, were then inserted into the neck muscles as electromyographic (EMG) electrodes. Successful insertion was confirmed when the movement frequency of the EMG wires was synchronized with respiration. The cortical, ground, and EMG electrodes were then assembled into a miniature socket and secured again with a thicker layer of glass ionomer cement. After surgery, mice that had not yet regained consciousness were placed on a heating blanket to maintain body temperature during recovery. The mice were then housed individually for 7 days of recovery and acclimated to the EEG recording environment for 24 h before formal monitoring.

### 2.8. EEG Recording

The head electrodes were connected to a multichannel swivel via the implanted recording cable and then linked to a PowerLab multichannel physiological recording system connected to a computer. EEG and EMG activities were recorded using LabChart 8.0 software. During recording, the EEG low-frequency filter was set to 0.5 Hz, the high-frequency filter was set to 50 Hz, and the sampling rate was set to 1000 Hz. After postoperative recovery and acclimation, continuous EEG/EMG recordings were performed for 8 h, with the same recording duration used for all groups.

### 2.9. EEG Analysis

Mouse EEG and EMG recordings were analyzed using the Lunion Stage automatic sleep analysis system. Sleep–wake states were scored in 10-s epochs and classified as wakefulness (WAKE), non-rapid eye movement sleep (NREM sleep), or rapid eye movement sleep (REM sleep). WAKE was characterized by low-amplitude, high-frequency EEG activity accompanied by prominent EMG activity; NREM sleep was characterized by high-amplitude, low-frequency EEG activity; reduced EMG activity; and predominant delta activity (0.5–4 Hz); and REM sleep was characterized by low-amplitude, high-frequency EEG activity; minimal EMG activity; and predominant theta activity (4–8 Hz). Before analysis, the EEG/EMG recordings were relabeled with anonymized codes that did not reveal group allocation. Sleep-stage scoring and data extraction were completed before the group identities were revealed. The automatic classifications were subsequently independently reviewed and corrected by two investigators, with inter-rater agreement exceeding 85%. For spectral analysis, delta power during NREM sleep (0.5–4 Hz) and theta power during REM sleep (4–8 Hz) were analyzed over the 8-h recording period.

### 2.10. Nissl and H&E Staining

After fixation in 4% paraformaldehyde, the brain tissue was embedded in paraffin and sectioned into 4 μm-thick slices. Sections were stained with 1% toluidine blue solution or hematoxylin and eosin solution after being dewaxed and rehydrated. H&E staining was used for the qualitative assessment of overall hippocampal tissue architecture and cellular arrangement, whereas Nissl staining was used for the qualitative examination of the distribution and staining characteristics of Nissl substance.

### 2.11. Enzyme-Linked Immunosorbent Assay (ELISA)

Mice were anesthetized by intraperitoneal injection of sodium pentobarbital (50 mg/kg), and serum and hippocampal tissues were collected for analysis. The levels of 5-hydroxytryptamine (5-HT), γ-aminobutyric acid (GABA), norepinephrine (NE), dopamine (DA), and glutamate (Glu) in hippocampal tissue, as well as the serum levels of interleukin-1β (IL-1β) and interleukin-18 (IL-18), were determined using commercially available ELISA kits (Jiangsu Meimian Industrial Co., Ltd., Jiangsu, China) according to the manufacturer’s instructions. The catalog numbers were as follows: 5-HT, MM-0443M1; GABA, MM-0442M1; NE, MM-0876M1; DA, KT3231-A; Glu, MM-44113M1; IL-1β, KT3070-A; and IL-18, KT2040-A. Hippocampal tissues were homogenized in ice-cold saline, centrifuged at 5000 rpm for 15 min at 4 °C, and the supernatants were collected for ELISA analysis. Serum samples were prepared in accordance with the manufacturer’s instructions. Standard curves were generated using the standards provided in the kits, and all samples were assayed in duplicate or triplicate.

### 2.12. RT-qPCR

Total RNA was extracted from mouse hippocampal tissues using the SteadyPure Universal RNA Extraction Kit (AG21017; Accurate Biotechnology Co., Ltd., Changsha, China). All RNA-related reagents and primers used for RT-qPCR were purchased from Accurate Biotechnology Co., Ltd. (Changsha, China). The primer sequences are listed in Table 1. RNA purity was determined spectrophotometrically, and an A260/A280 ratio between 1.8 and 2.2 indicated acceptable RNA quality. Reverse transcription was performed using Evo M-MLV RT Premix (AG11706). Quantitative PCR was then carried out on a real-time PCR system (C6, TargetingOne, Beijing, China) using the SYBR Green Premix Pro Taq HS qPCR Kit (AG11701) to determine the mRNA expression levels of the target genes. The PCR program consisted of 40 cycles of denaturation at 95 °C for 10 s, annealing at 60 °C for 10 s, and extension at 60 °C for 30 s. All procedures were performed strictly in accordance with the manufacturers’ instructions. The primers were synthesized by Beijing Dingguo Changsheng Biotechnology Co., Ltd. (Beijing, China). *β-actin* was used as the internal reference gene, and the relative expression levels of *NLRP3*, *ASC*, *caspase-1*, *GSDMD*, *IL-1β*, and *IL-18* in hippocampal tissue were determined. Relative gene expression was calculated using the 2^−ΔΔCt^ method.

### 2.13. Western Blot Analysis

Hippocampal tissues were collected from mice and fully lysed in RIPA lysis buffer. Total protein concentration was determined using the bicinchoninic acid (BCA) protein assay. After denaturation in SDS sample loading buffer, protein samples were separated by electrophoresis on 7.5% or 10% SDS-PAGE gels. Proteins were then transferred onto polyvinylidene fluoride (PVDF) membranes (IPVH00010, Merck Millipore, Arklow Ireland), which were blocked with 5% skim milk at room temperature for 1.5 h. The membranes were subsequently incubated overnight at 4 °C with the following primary antibodies: NLRP3 (rabbit monoclonal; Abcam, Cambridge, UK; ab263899; 1:1000), GSDMD, detecting both full-length and N-terminal fragments (rabbit monoclonal; Abcam, Cambridge, UK; ab219800; 1:1000), cleaved caspase-1/pro-caspase-1 (rabbit monoclonal; Abcam, Cambridge, UK; ab179515; 1:1000), ASC (rabbit monoclonal; Cell Signaling Technology, Danvers, MA, USA; 67824S; 1:1000), and β-actin (mouse monoclonal; Proteintech, Rosemont, IL, USA; 66009-1-Ig; 1:10,000). On the following day, the membranes were incubated with HRP-conjugated goat anti-rabbit and goat anti-mouse IgG (H + L) secondary antibodies (LABLEAD, Beijing, China; S0101 and S0100, respectively; both 1:5000) at room temperature for 1 h. Protein bands were visualized using an enhanced chemiluminescence (ECL) kit (SQ201, Epizyme Biomedical, Shanghai, China) and imaged using a Tanon 5200 imaging system (Tanon, Shanghai, China). Band intensities were quantified using ImageJ software (version 1.54p). For proteins analyzed as cleavage products, the relative levels were expressed as the ratios of GSDMD-N/GSDMD and cleaved caspase-1/pro-caspase-1. For the remaining target proteins, band intensities were normalized to β-actin. The β-actin bands shown in the representative Western blot images corresponded to the same sample set used for the indicated target proteins.

### 2.14. Statistical Analysis

All data were analyzed using SPSS 26.0, and graphs were generated using GraphPad Prism 9.0. Data normality was assessed using the Shapiro–Wilk test. Homogeneity of variances was assessed using the Brown–Forsythe test. For two-group comparisons, unpaired *t*-tests, Welch’s *t*-tests, or Mann–Whitney U tests were used, as appropriate. Normally distributed data from experiments involving more than two groups were analyzed using ordinary one-way ANOVA when variances were homogeneous, or Brown–Forsythe and Welch ANOVA tests when variances were unequal. Lognormally distributed data were analyzed using the corresponding lognormal procedures, whereas non-normally distributed data were analyzed using the Kruskal–Wallis test. For planned comparisons against the prespecified reference group, Dunnett’s test was used following ordinary one-way ANOVA, Dunnett’s T3 test following Brown–Forsythe and Welch ANOVA, and Dunn’s test following the Kruskal–Wallis test. The Control group was used as the reference in experiments without model induction, whereas the Model group was used as the reference in experiments with model induction. Data are expressed as the mean ± standard deviation (SD) unless otherwise indicated. A *p* value < 0.05 was considered statistically significant.

## 3. Results

### 3.1. Chemical Characterization and Quantitative Analysis of PVC

**Figure 1:**
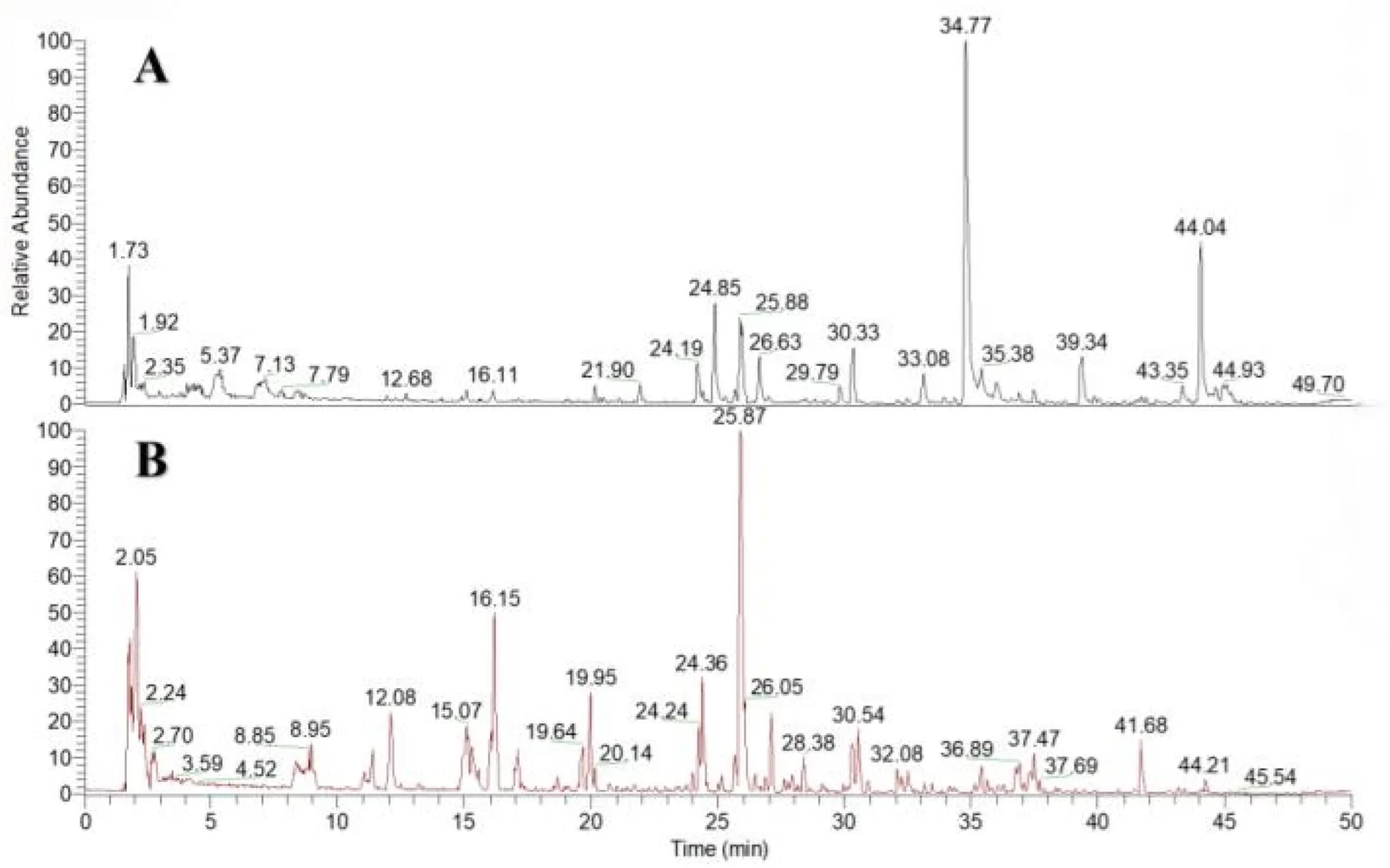
UPLC-MS-based chemical profile of PVC. (**A**) Representative base peak chromatogram of PVC in positive ion mode. (**B**) Representative base peak chromatogram of PVC in negative ion mode. PVC, Prunella vulgaris compound.

To characterize the chemical profile of PVC and provide a chemical basis for quality control, UPLC-Q-Exactive Orbitrap MS analysis was performed. Representative base peak chromatograms of PVC were obtained in both positive and negative ion modes (Figure 1A,B), with multiple detectable peaks observed across the 0–50 min analytical window.

Based on accurate mass measurements, MS/MS fragmentation patterns, and comparisons with reference standards, databases, or previously published data where available, a total of 198 representative chemical constituents were tentatively characterized in PVC. These constituents were classified into several categories, including flavonoids, phenolic acids and their derivatives, triterpenoids, sesquiterpenoids, alkaloids, amino acids, quinones, coumarins, lignans, fatty acids and related organic acids, and other compounds. Among them, flavonoids and phenolic acids and their derivatives accounted for a relatively large proportion of the tentatively characterized constituents.

Detailed information on the tentatively characterized constituents, including retention time, ion mode, observed *m*/*z*, molecular formula, fragment ions, compound category, and proposed identification, is provided in Supplementary Table S1. Targeted HPLC analysis was further performed to provide quantitative chemical information for the PVC batch used in the animal experiments. The contents of salviaflaside, rosmarinic acid, and linarin were 1.2412 ± 0.0253, 3.4656 ± 0.0514, and 4.3512 ± 0.0703 mg/g of the final PVC granules, respectively. The corresponding RSD values were 2.04%, 1.48%, and 1.62%, respectively (Supplementary Table S2). Together, the UPLC-MS profile and targeted HPLC quantification provide complementary qualitative and quantitative chemical information for the PVC batch used in the subsequent pharmacological experiments.

### 3.2. The PVC Exerts Sedative-Hypnotic Effects and Improves Sleep in Insomnia Model Mice

**Figure 2:**
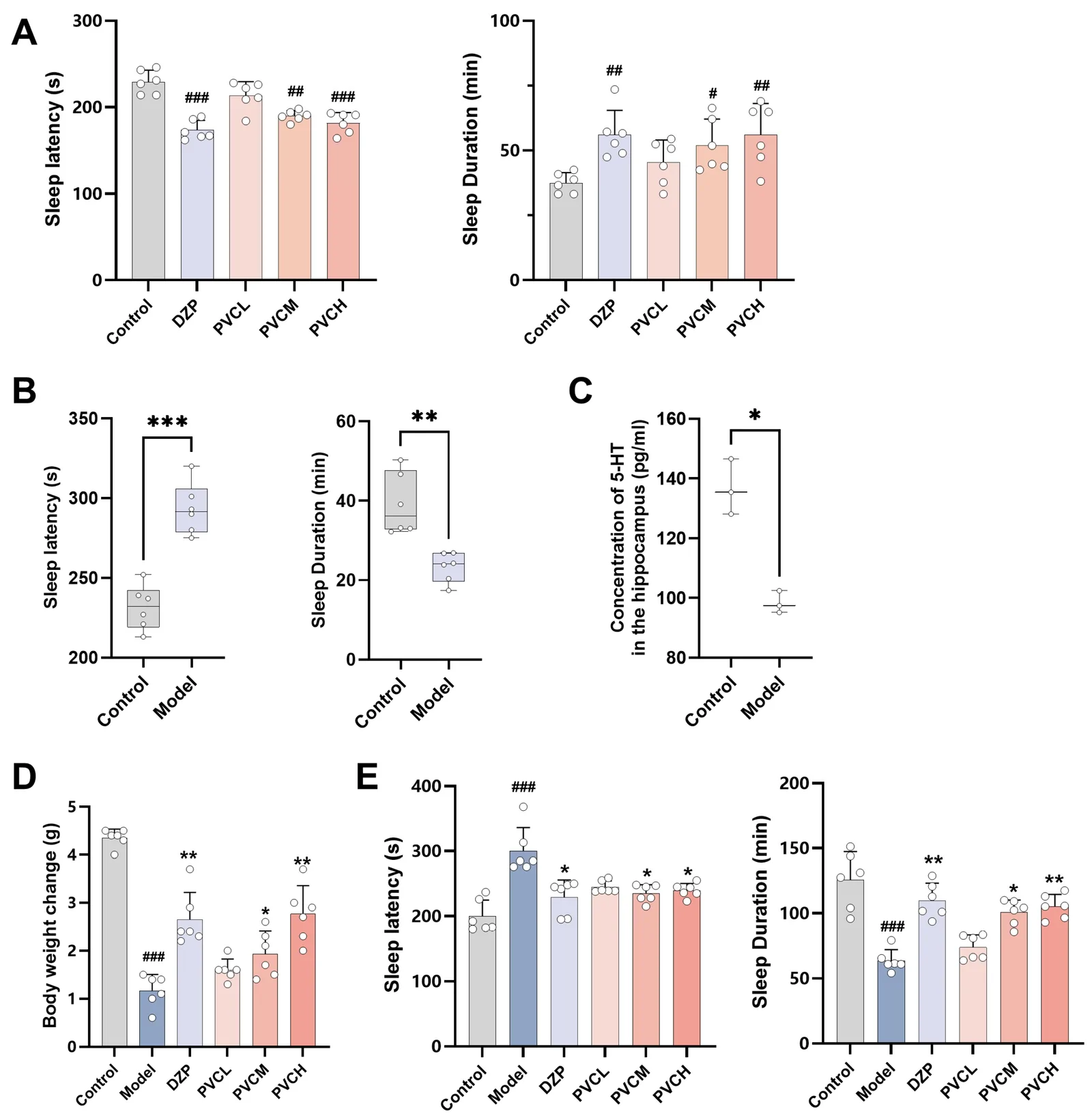
Sedative-hypnotic effects of PVC and its effects on sleep in insomnia model mice. Mice were treated with PVCL, PVCM, or PVCH (0.43, 0.86, and 1.72 g/kg, respectively) or diazepam (0.39 mg/kg) for 5 days. (**A**) The PIST was used to assess sleep latency and sleep duration, demonstrating the sedative-hypnotic effect of PVC. (**B**) PIST was performed to verify successful model establishment. (**C**) Hippocampal 5-HT levels were measured for model validation and were significantly reduced in the model group (*n* = 3 per group). (**D**) Body weight change (g), calculated as post-treatment body weight minus pretreatment body weight. (**E**) Sleep latency and sleep duration after treatment in each group. Data are presented as the mean ± SD (*n* = 6 per group; panel (**C**), *n* = 3). Each white circle represents an individual animal. # *p* < 0.05, ## *p* < 0.01, ### *p* < 0.001 vs. control group; * *p* < 0.05, ** *p* < 0.01, *** *p* < 0.001 vs. model group.

The PIST is an important experimental method for evaluating the sedative-hypnotic effects of drugs. In this assay, mice are placed into a sedated state using a hypnotic agent, and sleep latency and sleep duration are then assessed. In the present study, compared with the control group, the PVC significantly shortened sleep latency and prolonged sleep duration in mice. Notably, the effect of the PVCH group was comparable to that of the diazepam group (Figure 2A), indicating a pronounced sedative-hypnotic effect.

To further verify its therapeutic efficacy in insomnia, a PCPA-induced insomnia model was established. As an inhibitor of tryptophan hydroxylase, PCPA markedly depletes 5-HT, thereby disrupting the sleep–wake rhythm. Mice in the model group exhibited typical insomnia-like manifestations, including increased daytime activity, abnormal behaviors such as crowding and a tendency to fight, and circadian rhythm disturbance. In addition, hippocampal 5-HT levels were significantly reduced in a subset of animals used for model validation (Figure 2C), providing supportive evidence for successful model establishment. Compared with the control group, the model group showed significantly prolonged sleep latency and markedly shortened sleep duration (Figure 2B), which is consistent with the classical features of insomnia. Following intervention with the PVC, these abnormalities were significantly reversed, as evidenced by shortened sleep latency and prolonged sleep duration, indicating a favorable therapeutic effect (Figure 2E).

Body weight change from before to after treatment was also evaluated in the insomnia-model experiment. Body weight change was significantly lower in the model group, whereas diazepam, PVCM, and PVCH significantly attenuated this reduction (Figure 2D). This change may be associated with appetite and metabolic disturbances caused by insufficient sleep, which in turn affect body weight. These findings suggest a potential association between sleep improvement and body weight regulation. Based on the pharmacodynamic results, PVCM, which represented an effective intermediate dose, was selected as the typical sample for the PVC intervention group in the subsequent related experiments.

Collectively, these results demonstrate that the PVC not only exerts definite sedative-hypnotic effects in normal mice, but also significantly ameliorates sleep-onset difficulty and insufficient sleep in the induced insomnia model.

### 3.3. The PVC Improves Sleep Architecture in Insomnia Model Mice and Promotes the Recovery of REM Sleep

To further elucidate the role of the PVC in sleep regulation, EEG and EMG were used to precisely classify and dynamically monitor sleep stages in mice. As the gold standard for sleep assessment, EEG/EMG allows clear discrimination among WAKE, NREM sleep, and REM sleep, thereby providing a direct representation of sleep architecture.

**Figure 3:**
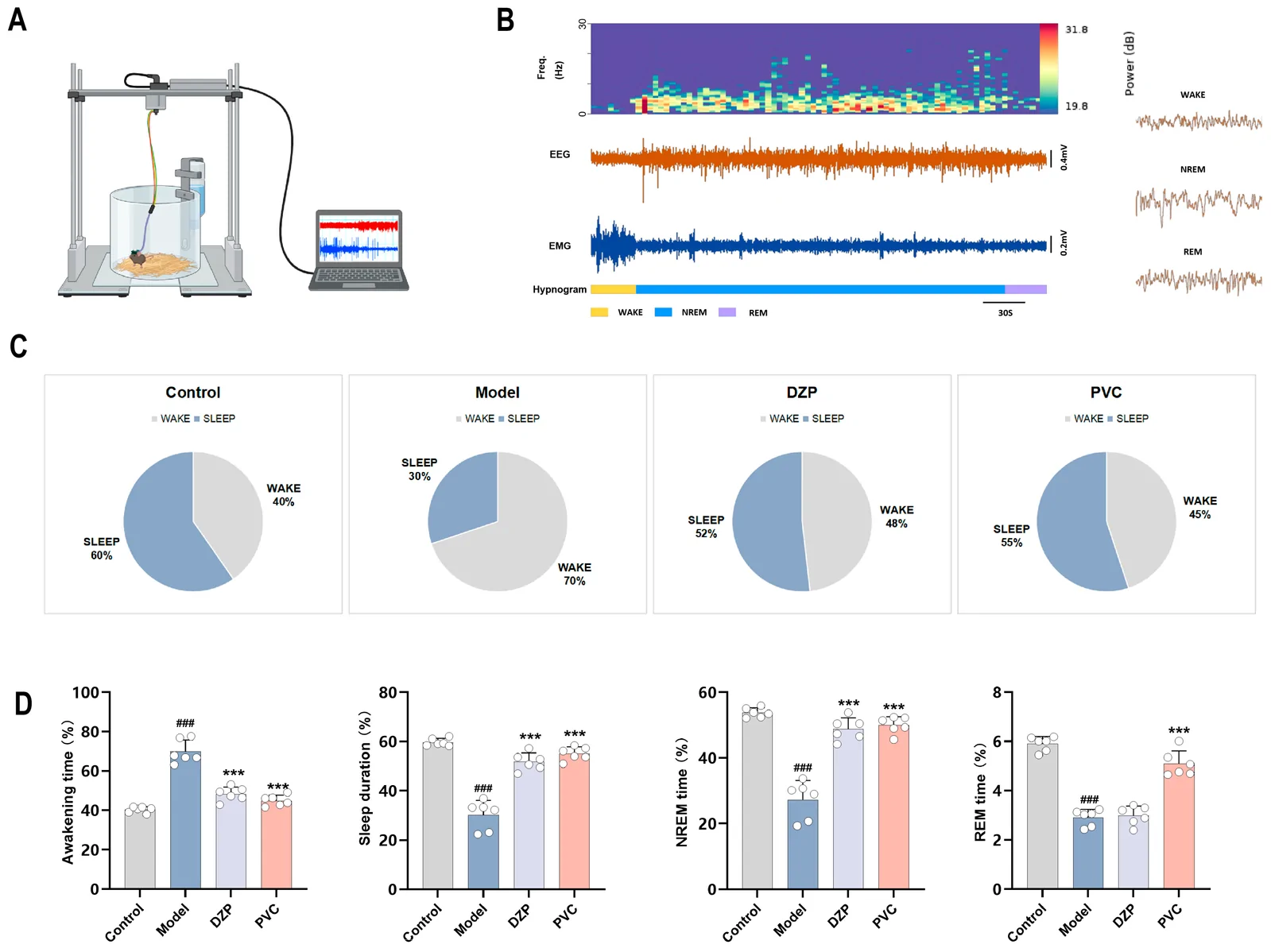
EEG-based analysis of sleep-stage distribution among the different groups. (**A**) Schematic diagram of the EEG recording setup. (**B**) Representative EEG waveforms for sleep-stage classification. (**C**) Pie charts showing the proportions of wakefulness and sleep in each group. (**D**) Bar graphs showing the time spent in wakefulness, NREM sleep, and REM sleep in each group. The white circles represent individual animals. This has been clarified in the figure legend. ### *p* < 0.001 vs. control group; *** *p* < 0.001 vs. model group.

As shown in the sleep proportion analysis (Figure 3C), compared with the control group (wakefulness, 40%; sleep, 60%), the model group exhibited a marked increase in wakefulness to 70% and a decrease in sleep to 30%. Following diazepam treatment, the proportion of wakefulness decreased to 48% and sleep increased to 52%, whereas in the PVC group, wakefulness decreased to 45% and sleep recovered to 55%, showing recovery of the total sleep proportion in both treatment groups.

Further analysis (Figure 3D) showed that the model group displayed significantly prolonged wake time and markedly reduced total sleep time, NREM sleep time, and REM sleep time. Both diazepam and the PVC significantly reduced wake time and prolonged total sleep time and NREM sleep time. Notably, the observed increase in REM sleep time was more pronounced in the PVC group under the present experimental conditions. These findings indicate that both treatments improved overall sleep architecture, with differences in the extent of REM sleep recovery under the present experimental conditions.

**Figure 4:**
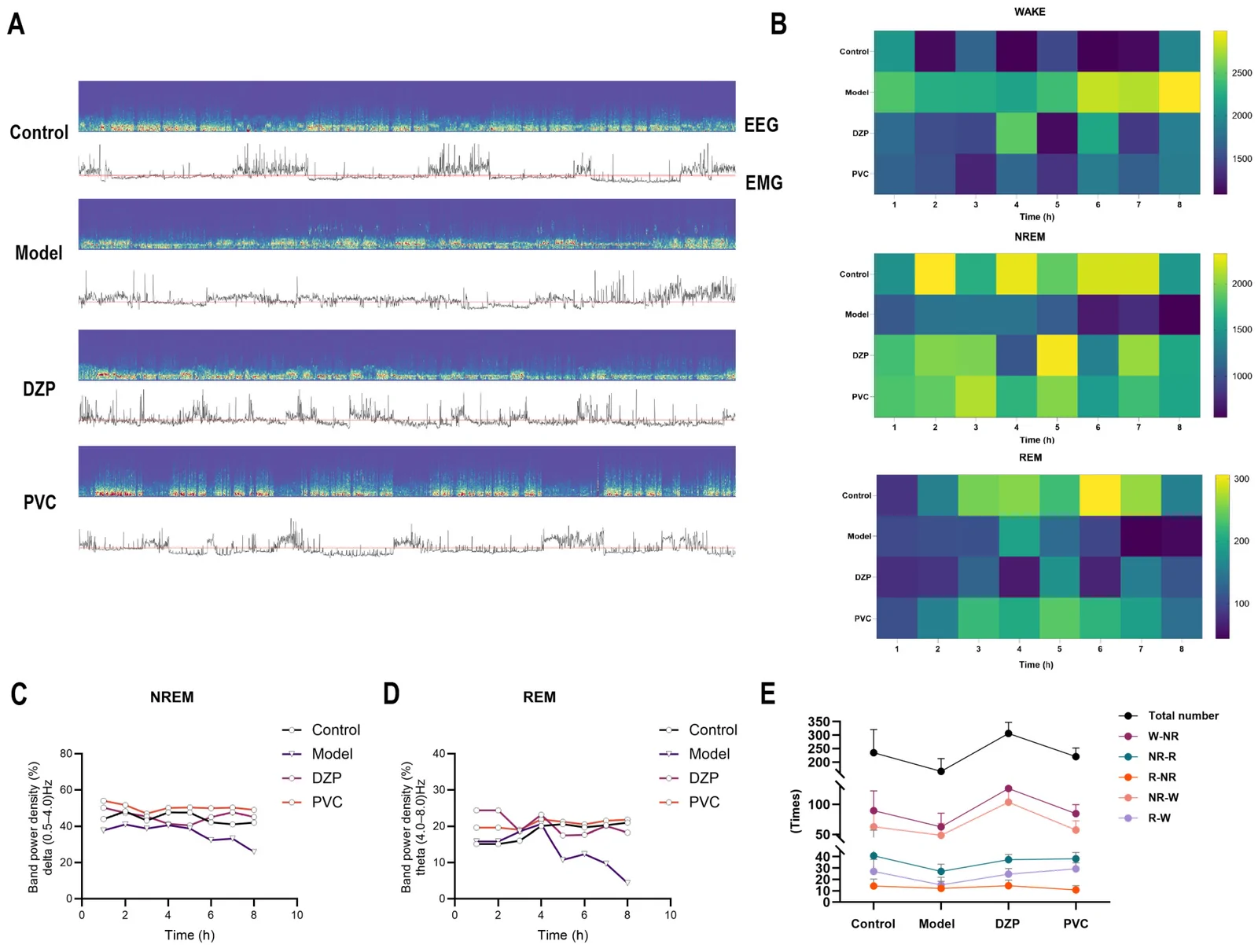
EEG-based sleep analysis in each group (*n* = 6). (**A**) Representative EEG and EMG recordings. In panel (**A**), the colored spectrograms represent EEG spectral power, the black traces represent EMG signals, and the red horizontal lines indicate the zero-amplitude baseline. (**B**) Sleep-stage heatmaps showing the temporal distribution of wakefulness, NREM sleep, and REM sleep. (**C**) Time-course curves of delta power during NREM sleep. (**D**) Time-course curves of theta power during REM sleep. (**E**) Numbers of transitions between different sleep stages. W–NR, WAKE-to-NREM; NR–R, NREM-to-REM; R–NR, REM-to-NREM; NR–W, NREM-to-WAKE; R–W, REM-to-WAKE.

The time-course curve of delta power during NREM sleep (0.5–4 Hz, Figure 4C) showed that power was significantly reduced in the model group, whereas both the PVC and diazepam groups exhibited marked recovery with relatively stable restoration curves. Similarly, the time-course curve of theta power during REM sleep (4–8 Hz, Figure 4D) showed a marked reduction throughout the recording period in the model group, whereas both treatment groups showed partial recovery.

### 3.4. The PVC Regulates Sleep Architecture and Improves Sleep Quality in Insomnia Model Mice

To further elucidate the effects of the PVC on sleep quality, we analyzed the distribution, power changes, and transition patterns of WAKE, NREM sleep, and REM sleep. These parameters are key indicators for evaluating the integrity and quality of sleep homeostasis.

EEG traces (Figure 4A) showed regular sleep waveforms in the control group, reflecting normal sleep homeostasis and rhythmicity. In contrast, the model group exhibited disorganized EEG patterns, disrupted sleep rhythms, markedly shortened sleep time, and severely disturbed sleep homeostasis. In the treatment groups, EEG patterns showed recovery toward normal; representative EEG traces in the PVC group displayed relatively regular waveforms and a sleep pattern more closely resembling that of the control group, whereas the diazepam group retained a less regular waveform organization. These observations suggest that the two treatments produced different patterns of sleep organization.

Sleep heatmap analysis (Figure 4B) showed that, over the 8 h recording period, WAKE, NREM, and REM stages were evenly distributed in the control group, reflecting a natural sleep–wake rhythm. In contrast, the model group was dominated by WAKE, whereas NREM and REM episodes were sparse and fragmented, indicating severely disrupted sleep architecture. Both diazepam and the PVC promoted recovery of the sleep pattern; however, the NREM and REM episodes in the PVC group were more continuous and regular, more closely approximating the physiological state of the control group, indicating differences in sleep continuity between the two treatment groups.

In sleep studies, analysis of sleep-stage transitions is critical for assessing sleep fragmentation and pharmacological efficacy (Figure 4E). In the model group, the total number of transitions and specific transitions between stages, such as WAKE-to-NREM, NREM-to-REM, and REM-to-WAKE, were markedly reduced, reflecting prolonged wakefulness and disrupted sleep continuity. Both diazepam and the PVC significantly increased the number of transitions, thereby improving sleep continuity. Specifically, the diazepam group showed significantly increased WAKE-to-NREM and NREM-to-WAKE transitions, even exceeding those in the control group, whereas the PVC group exhibited a more moderate increase in transition frequency. These findings indicate that diazepam and PVC produced distinct sleep-stage transition profiles.

Collectively, these results indicate that the PVC attenuates PCPA-induced disturbances in sleep architecture and shifts the proportions of WAKE, NREM sleep, and REM sleep toward those observed in the control group.

### 3.5. Effects of PVC on Hippocampal Histomorphology and Neurotransmitter Levels in Insomnia Model Mice

**Figure 5:**
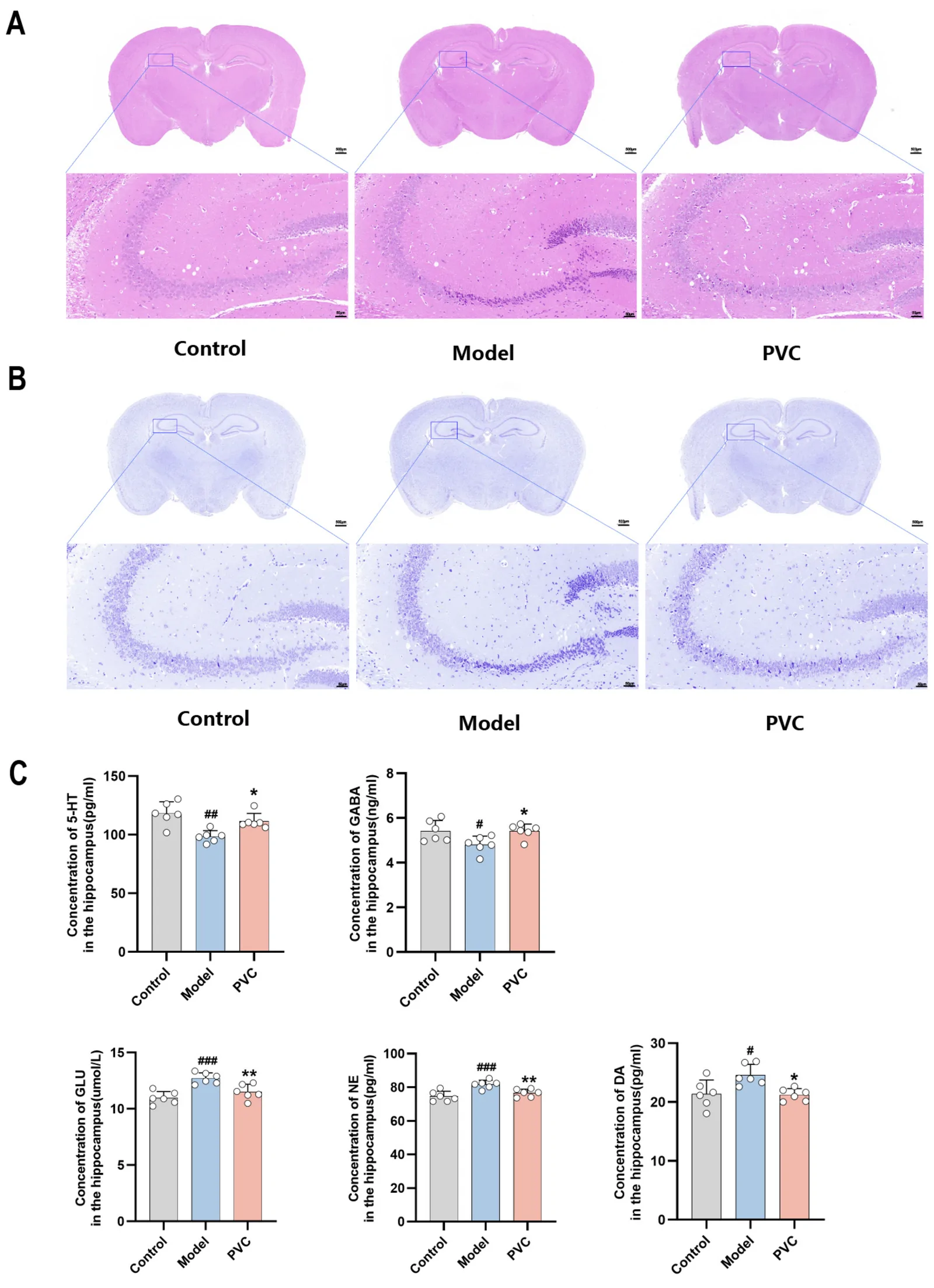
Representative histological images of the hippocampus and neurotransmitter levels in hippocampal tissue (*n* = 5). (**A**) Representative H&E staining of the hippocampal region in each group, including an overview and a magnified view of the boxed region. (**B**) Representative Nissl staining of the hippocampal region in each group, including an overview and a magnified view of the boxed region. Scale bars: 500 μm for the overview images and 50 μm for the magnified images. (**C**) Neurotransmitter levels in hippocampal tissue in each group (*n* = 6) Each white circle represents an individual animal. # *p* < 0.05, ## *p* < 0.01, ### *p* < 0.001 vs. control group; * *p* < 0.05, ** *p* < 0.01, vs. model group.

H&E-stained sections of the hippocampal CA3 region were examined microscopically in each group (Figure 5A). In the control group, hippocampal cells were relatively regularly arranged, with preserved overall tissue organization. In contrast, cells in the hippocampal CA3 region of the model group showed less regular arrangement and altered tissue organization. Following treatment with the PVC, hippocampal cellular arrangement appeared comparatively more regular, with clearer tissue organization, suggesting that the compound was associated with less pronounced PCPA-induced histomorphological alterations in the hippocampus.

Further Nissl staining results showed that, in the model group, the Nissl substance appeared more lightly stained and less regularly distributed compared with the control group (Figure 5B), altered Nissl staining characteristics. By contrast, Nissl staining in the PVC-treated group showed comparatively clearer staining and more regular distribution, suggesting less pronounced histomorphological alterations in the hippocampus.

To further investigate the underlying mechanism, we also measured neurotransmitter levels in the hippocampus of insomnia model mice (Figure 5C). Compared with the control group, the concentrations of the inhibitory neurotransmitters 5-HT and GABA were significantly reduced in the model group, whereas the levels of the excitatory neurotransmitters Glu and DA were significantly increased, suggesting that insomnia induced neurotransmitter imbalance. After treatment with the PVC, the levels of 5-HT and GABA were significantly increased, whereas those of Glu and DA were significantly decreased, with particularly prominent changes in GABA and Glu, indicating that the compound may contribute to the improvement in insomnia-like phenotypes, at least in part, by modulating neurotransmitter homeostasis.

Based on the EEG findings, insomnia markedly disrupted sleep architecture, particularly by reducing the proportion of REM sleep. The histological findings provided qualitative and supportive evidence of hippocampal morphological alterations in the model group, as reflected by less regular cellular arrangement and weaker Nissl staining. Together with the partial recovery of REM sleep after treatment, these observations were consistent with less pronounced insomnia-associated hippocampal pathological alterations following PVC treatment.

### 3.6. The PVC Attenuates Neuroinflammation and Reduces NLRP3 Inflammasome- and Pyroptosis-Related Markers in Insomnia Model Mice

**Figure 6:**
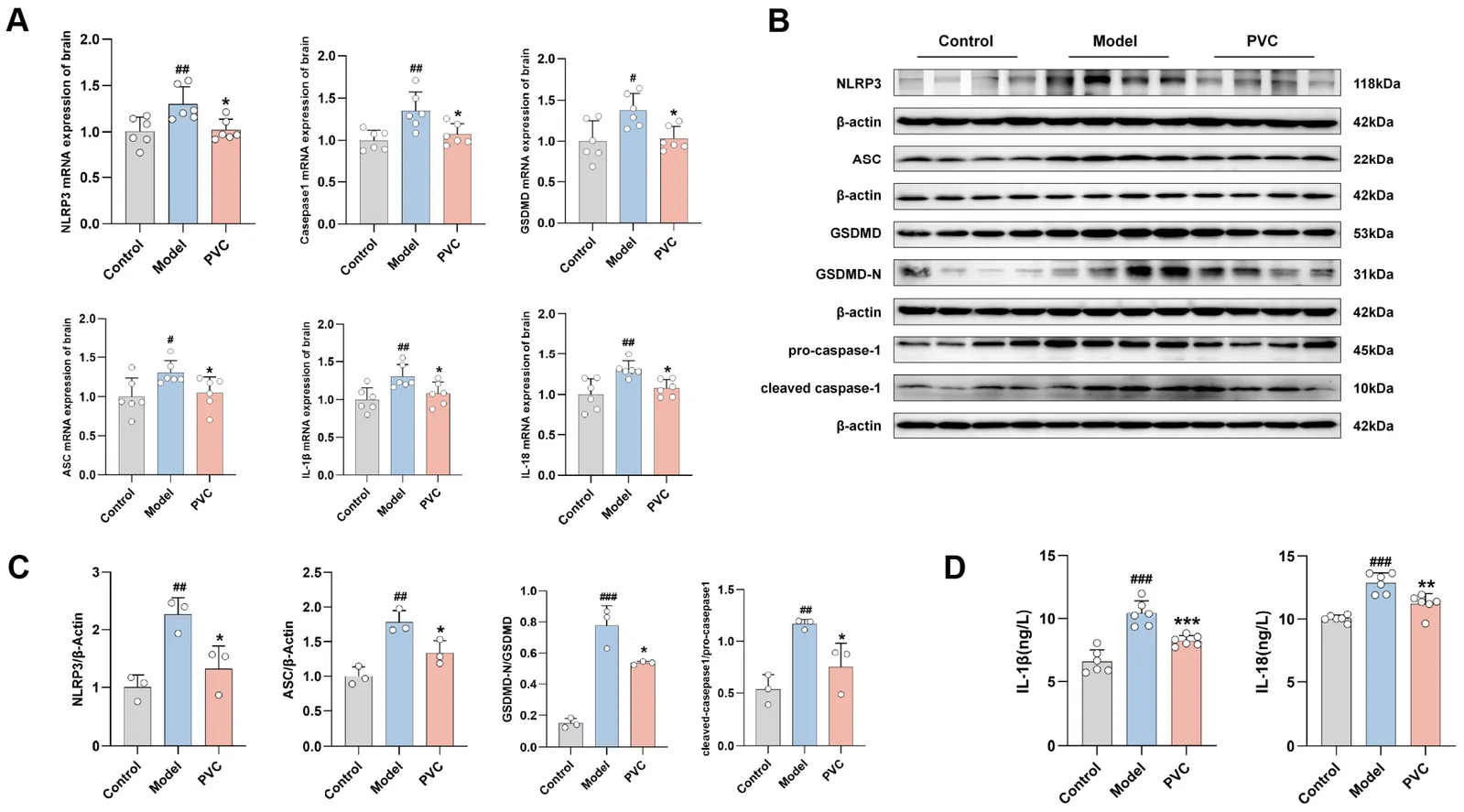
RT-qPCR and Western blot analyses of the NLRP3 inflammasome-mediated pyroptotic pathway. (**A**) RT-qPCR analysis of pyroptosis-related genes (*n* = 6 per group). (**B**) Representative Western blot images of pyroptosis-related proteins (*n* = 3 per group). (**C**) Quantitative analysis of Western blot results (*n* = 3). (**D**) ELISA analysis of downstream inflammatory cytokines (*n* = 6 per group). Each white circle represents an individual animal. # *p* < 0.05, ## *p* < 0.01, ### *p* < 0.001 vs. control group; * *p* < 0.05, ** *p* < 0.01, *** *p* < 0.001 vs. model group.

We first evaluated the mRNA expression levels of *NLRP3*, *ASC*, *caspase-1*, *GSDMD*, *IL-1β*, and *IL-18* in hippocampal tissues from each group by RT-qPCR (Figure 6A). The model group showed increased mRNA expression of these NLRP3 inflammasome- and pyroptosis-related molecules. In contrast, PVC treatment reduced the mRNA expression levels of *NLRP3*, *ASC*, *caspase-1*, *GSDMD*, *IL-1β*, and *IL-18*, indicating an association between PVC treatment and lower expression of these pathway-related markers.

We next examined proteins related to canonical NLRP3/caspase-1/GSDMD signaling. Compared with the model group, the PVC group showed reduced expression of NLRP3, ASC, pro-caspase-1, GSDMD, and GSDMD-N, as well as reduced cleaved caspase-1 levels according to the defined normalization strategy (Figure 6B,C). In particular, the GSDMD-N/GSDMD and cleaved caspase-1/pro-caspase-1 ratios were decreased after PVC treatment, consistent with reduced pyroptosis-related molecular signaling.

Finally, ELISA was used to determine the levels of pyroptosis-related inflammatory cytokines. As shown in Figure 6D, serum levels of inflammatory cytokines were markedly elevated in the model group, whereas treatment with the PVC significantly reduced the levels of these inflammation-related factors in insomnia model mice.

Collectively, these results show that PVC treatment was associated with lower levels of NLRP3 inflammasome- and pyroptosis-related markers and a reduced inflammatory response in insomnia model mice.

## 4. Discussion

This study investigated the effects of PVC on insomnia-like phenotypes and the associated neurochemical and inflammatory changes. Our results showed that, in a PCPA-induced mouse model of insomnia, PVC significantly shortened sleep latency; prolonged sleep duration; improved sleep architecture, including increased REM sleep; attenuated hippocampal histomorphological alterations; modulated neurotransmitter levels; and reduced NLRP3 inflammasome- and pyroptosis-related markers together with the associated inflammatory response.

These findings highlight the multilevel sleep-regulatory properties of the PVC. The shortening of pentobarbital-induced sleep latency and prolongation of sleep duration suggest that the compound may enhance sleep-promoting processes, which is consistent with the observed increases in hippocampal 5-HT and GABA levels and decreases in Glu and DA levels after treatment. Previous studies have shown that GABA-related interventions primarily shorten sleep latency by enhancing overall sleep-promoting effects; however, evidence for their specific regulation of REM sleep remains limited [28]. Likewise, previous studies on *Prunella vulgaris* and its active constituents have largely focused on sedative-hypnotic-like effects, whereas their ability to modulate sleep architecture and attenuate insomnia-associated hippocampal pathological alterations has remained poorly understood [29].

However, the regulation of sleep architecture, especially REM sleep, is multifactorial and cannot be fully explained by changes in a single neurotransmitter system. Under the present experimental conditions, PVC was associated with a relatively balanced recovery of sleep-stage distribution, particularly REM sleep, whereas diazepam produced a more pronounced increase in wake–sleep transitions. These findings suggest that the two treatments may differentially affect sleep architecture. However, because the study was not designed for a direct superiority comparison, these differences should be interpreted cautiously. Therefore, the observed changes in REM sleep may be associated with broader changes in neurotransmitter balance, hippocampal histomorphology, and neuroinflammatory markers rather than simple enhancement of central inhibition.

In recent years, pyroptosis has been recognized as a potential contributor to sleep-disturbance-associated neuroinflammatory injury. This process is typically characterized by excessive activation of the NLRP3 inflammasome, which triggers caspase-1-dependent cleavage of GSDMD and the release of inflammatory cytokines such as IL-1β and IL-18, thereby contributing to neuroinflammatory and cellular injury. In the hippocampus of insomnia model mice, we observed significant upregulation of NLRP3, ASC, caspase-1, GSDMD, and its cleavage product GSDMD-N, accompanied by increased levels of IL-1β and IL-18, a molecular pattern consistent with enhanced NLRP3 inflammasome- and pyroptosis-related signaling. Following treatment with the PVC, the levels of these proteins and inflammatory mediators were reduced, supporting an association between PVC treatment and attenuation of these molecular and inflammatory responses.

**Figure 7:**
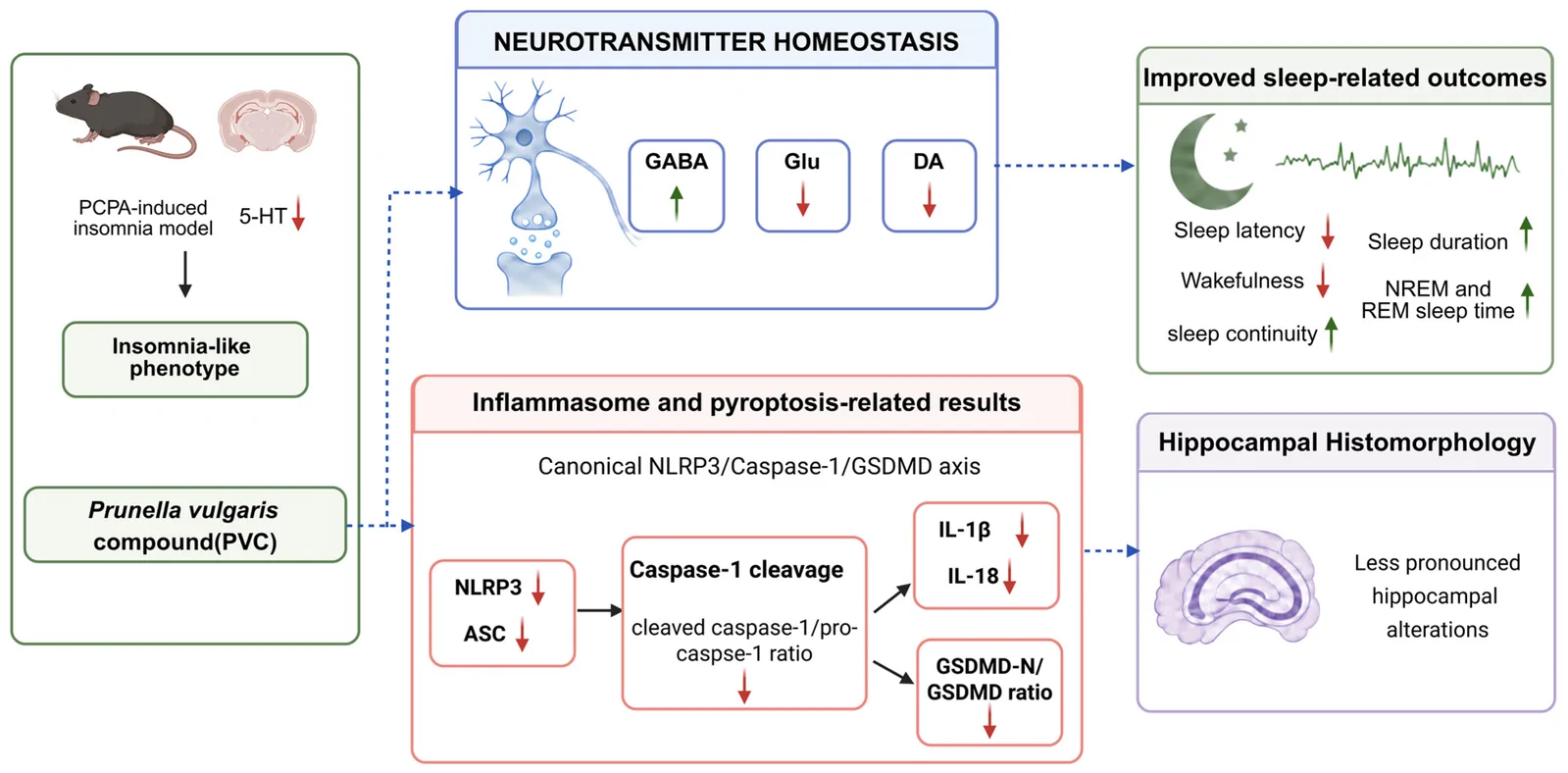
Proposed working model summarizing the observed findings and proposed associations following Prunella vulgaris compound (PVC) treatment in PCPA-induced insomnia model mice. Solid black arrows indicate established canonical pathway relationships, whereas blue dashed arrows indicate proposed associations based on the present findings; red downward and green upward arrows indicate decreases and increases, respectively. The decrease in 5-HT represents the model group relative to the control group and was used only for model validation, whereas the other directional arrows represent changes after PVC treatment relative to the model group. Hippocampal histomorphological findings were qualitative and supportive.

Although NLRP3/caspase-1/GSDMD-related signaling was the main molecular focus of this study, the present experiments measured changes in pathway-related markers but did not directly test causality. As a multi-component herbal formulation, the PVC may influence insomnia-related pathological changes through multiple biological processes, including neurotransmitter regulation, oxidative stress, circadian rhythm modulation, hypothalamic–hippocampal communication, and broader neuroimmune interactions. Because no pathway-specific inhibitor, genetic manipulation, or rescue experiment was performed, the reductions in NLRP3 inflammasome- and pyroptosis-related markers should be interpreted as correlative evidence supporting pathway involvement rather than proof that this pathway causally mediates the effects of PVC. Future studies using pathway-specific inhibitors, genetic models, or cell-type-specific analyses will be necessary to establish a direct causal relationship between NLRP3-mediated pyroptosis and the observed effects of the PVC. A proposed working model summarizing the observed findings and proposed associations is shown in Figure 7.

The present work provides preclinical evidence linking the sleep-related effects of PVC with changes in neurotransmitter and inflammation-related markers. As a multicomponent herbal formulation, PVC warrants further investigation in sleep disorders. Future studies should incorporate chronic insomnia models and the identification of active constituents to further clarify long-term efficacy and optimize dosing strategies.

This study has several limitations. First, only an acute PCPA-induced insomnia model was used, which cannot fully recapitulate the multifactorial nature of chronic insomnia in humans. Second, different mouse strains were used for different experimental endpoints, which may introduce strain-related variability in sleep parameters or treatment responses; therefore, the findings should be interpreted within their respective experimental systems. Third, some assays involved relatively small sample sizes, particularly the Western blot analysis and the preliminary 5-HT measurement used for model validation (both *n* = 3 per group). Fourth, the histological findings were based mainly on representative H&E and Nissl staining without comprehensive quantitative morphometric analysis. Therefore, these histological observations should be regarded as qualitative and supportive evidence and further validated in larger cohorts with quantitative histological assessment, chronic insomnia models, and translational studies. Because this study did not include behavioral tests of learning, memory, or emotional function, the relationship between PVC-mediated improvements in sleep architecture and potential cognitive or affective benefits remains to be investigated in future studies.

In conclusion, this study provides experimental evidence that PVC improves insomnia-like phenotypes and attenuates hippocampal pathological alterations in PCPA-induced insomnia model mice. These effects may be associated with the restoration of neurotransmitter balance and sleep architecture, as well as reduced NLRP3/caspase-1/GSDMD-related signaling. UPLC-MS-based chemical characterization combined with targeted HPLC quantification provides preliminary chemical quality-control information for the PVC preparation used in this study. These findings provide supportive molecular and chemical evidence for further investigation of PVC in sleep disorders. Future studies should evaluate its active constituents, long-term efficacy, and translational relevance.

## Figures and Tables

**Table 1:** Primer sequence.

Gene Name	F-Terminal Sequence	R-Terminal Sequence
*NLRP3*	ATTGCTGTGTGTGGGACTGAA	ATCCTGACAACACGCGGAT
*ASC*	CTTACAGGAGCTGGCTGAGCA	GACCCTGGCAATGAGTGCTTG
*Caspase-1*	ATTGCTTTCTGCTCTTCAACACC	ACCAGGCATATTCTTTCATGTGT
*GSDMD*	AACCCCGTTATTCATGTGTCA	CAAAACACTCCGGTTCTGGT
*IL-1β*	AGACAACTGCACTACAGGCT	TTGTCGTTGCTTGGTTCTCCTT
*IL-18*	GCGTCAACTTCAAGGAAATGATGT	TGTCAACGAAGAGAACTTGGTCAT
*β-actin*	CATCCGTAAAGACCTCTATGCCAAC	ATGGAGCCACCGATCCACA

## Data Availability

The original contributions presented in this study are included in the article/Supplementary Materials. Further inquiries can be directed to the corresponding authors.

## Supplementary Materials

The following supporting information can be downloaded at: https://www.mdpi.com/article/10.3390/life16081268/s1, Table S1: Representative chemical constituents tentatively characterized in PVC by UPLC-Q-Exactive Orbitrap MS. Table S2: Quantitative determination of three selected chemical markers in the PVC batch used for the animal experiments.
