# Supplementary material for: Prunella vulgaris Compound Improves Sleep Architecture and Attenuates Hippocampal Injury in Insomnia Mice
Source: Life (Basel). 2026 Jul 31;16(8):1268. doi: 10.3390/life16081268 (PMC13514870; doi:10.3390/life16081268)
Supplement: Supplementary file 1 [file life-16-01268-s001.zip › Table S1.pdf]

Table S1. Representative chemical constituents tentatively characterized in PVC by UPLC-Q-Exactive Orbitrap MS.

Compound	Metabolites	Formula	theoreticalm/z	m/z	MassError (ppm)	Retentiontime (min)
1	Threonic acid	$C_4H_8O_5$	135.0298	135.0284	-0.370	0.11
2	3,4,5-Trimethoxytoluene	$C_{10}H_{14}O_3$	183.1015	183.1011	-0.401	0.49
3	Vanillic acid	$C_8H_8O_4$	167.0349	167.0328	-1.075	0.67
4	Diethyl sulfone	$C_4H_{10}O_2S$	123.0474	123.0436	-3.827	1.26
5	3-Hydroxybenzaldehyde	$C_7H_6O_2$	121.0295	121.0274	-0.966	1.44
6	Quinic acid	$C_7H_{12}O_6$	191.0561	191.0541	-0.845	1.72
7	Tartaric acid	$C_4H_6O_6$	149.0091	149.0091	-0.484	1.85
8	Malic acid	$C_4H_6O_5$	133.0142	133.0121	-1.000	1.81
9	Gluconate	$C_6H_{12}O_7$	195.0510	195.0491	-0.799	1.87
10	L-Pyroglutamic acid	$C_5H_7NO_3$	128.0353	128.0332	-0.970	2.03
11	Maleic acid	$C_4H_4O_4$	115.0036	115.0018	-0.745	2.06
12	Phenylalanine	$C_9H_{11}NO_2$	166.0862	166.0853	-0.925	2.08
13	Cystine	$C_6H_{12}N_2O_4S_2$	239.0165	239.0160	2.565	2.09
14	Citric acid	$C_6H_8O_7$	191.0197	191.0177	-0.869	2.15
15	Proline	$C_5H_9NO_2$	116.0706	116.0702	-0.365	2.32
16	Salvianic acid A	$C_9H_{10}O_5$	197.0455	197.0436	-0.840	2.34
17	1,2,4-Benzenetriol	$C_6H_6O_3$	125.0244	125.0224	-0.911	2.59
18	Succinic acid	$C_4H_6O_4$	117.0193	117.0172	1.445	3.11
19	2-Hydroxyphenyl formate	$C_7H_6O_3$	137.0244	137.0224	-0.871	3.36
20	Adenosine	$C_{10}H_{13}N_5O_4$	268.1040	268.1022	-1.800	3.74
21	L-Leucine	$C_6H_{13}NO_2$	130.0873	130.0851	-1.085	3.91
22	L-Isoleucine	$C_6H_{13}NO_2$	132.1019	132.1014	-0.445	4.2
23	Guanosine	$C_{10}H_{13}N_5O_5$	284.0989	284.0981	-0.835	4.33
24	Protocatechualdehyde	$C_7H_6O_3$	137.0244	137.0224	-0.891	4.41

Compound	Metabolites	Formula	theoreticalm/z	m/z	MassError (ppm)	Retentiontime (min)
25	Methyl danshensu	$C_{10}H_{12}O_5$	211.0611	211.0579	-2.190	4.81
26	Salicylic acid	$C_7H_6O_3$	137.0244	137.0224	-0.831	4.81
27	m-Hydroxybenzoic acid	$C_7H_6O_3$	137.0244	137.0224	-0.861	5.02
28	p-Hydroxybenzoic acid	$C_7H_6O_3$	137.0244	137.0224	-0.861	5.02
29	Esculetin	$C_9H_6O_4$	177.0193	177.0166	-1.545	5.58
30	2,6-Dimethoxy-p-benzoquinone	$C_8H_8O_4$	169.0495	169.0484	-1.135	6.46
31	2-Methoxycinnamic acid	$C_{10}H_{10}O_3$	177.0557	177.0539	-0.681	7.75
32	Protocatechuic acid	$C_7H_6O_4$	153.0193	153.0173	-0.885	8.71
33	Syringic acid	$C_9H_{10}O_5$	197.0455	197.0435	-0.870	8.89
34	Ethenone	$C_{11}H_{14}O_4$	211.0964	211.0937	-2.755	9.65
35	Tyramine	$C_8H_{11}NO$	138.0913	138.0904	-0.861	10.74
36	Caffeic acid	$C_9H_8O_4$	179.0349	179.0350	1.135	11.15
37	Dihydrocaffeic acid	$C_9H_{10}O_4$	181.0506	181.0486	-0.845	12.30
38	Tryptophan	$C_{11}H_{12}N_2O_2$	205.0971	205.0964	-0.744	12.57
39	Dihydroisoferulic acid-3-O-β-D-glucuronide	$C_{16}H_{20}O_{10}$	371.0983	371.0983	-5.533	12.65
40	Caffeic acid 3-O-glucoside	$C_{15}H_{18}O_9$	341.0878	341.084	-1.809	13.61
41	Chlorogenic acid	$C_{16}H_{18}O_9$	353.0878	353.0858	-0.829	13.89
42	Shikimic acid	$C_7H_{12}O_5$	175.0611	175.0593	-0.770	13.89
43	Isopropylmalic acid	$C_7H_{12}O_5$	175.0611	175.0599	-0.880	14.1
44	Cinnamic acid	$C_9H_8O_2$	147.0451	147.0434	-0.586	14.17
45	Mangiferin	$C_{19}H_{18}O_{11}$	421.0776	421.0784	1.892	14.39
46	Jaceosidin	$C_{17}H_{14}O_7$	329.0666	329.0648	-0.759	14.50
47	Caffeic acid 4-O-glucoside	$C_{15}H_{18}O_9$	341.087	341.0863	-0.379	14.54
48	Acetovanillone	$C_9H_{10}O_3$	167.0702	167.0692	-1.061	14.65
49	Ethyl p-coumarate	$C_{11}H_{12}O_3$	193.0859	193.0833	-2.561	14.68
50	Syringic acid	$C_9H_{10}O_5$	199.0600	199.0600	-3.050	15.09

Compound	Metabolites	Formula	theoreticalm/z	m/z	MassError (ppm)	Retentiontime (min)
51	3-O-Feruloylquinic acid	$C_{17}H_{20}O_9$	367.1034	367.1013	-1.039	15.19
52	Daphnetin	$C_9H_6O_4$	177.0193	177.0173	-0.905	15.6
53	Cyclomethyltryptophan	$C_{12}H_{12}N_2O_2$	217.0971	217.0959	-1.174	15.67
54	Coumaroyl hexoside	$C_{15}H_{18}O_8$	325.0928	325.0911	-0.604	15.85
55	Cryptochlorogenic acid	$C_{16}H_{18}O_9$	353.087	353.0853	-1.319	15.88
56	Scopoletin	$C_{10}H_8O_4$	191.0349	191.0331	-0.775	16.44
57	Dihydroferulic acid	$C_{10}H_{12}O_4$	195.0662	195.0643	-0.864	16.53
58	Asaraldehyde	$C_{10}H_{12}O_4$	197.0808	197.0814	0.605	16.70
59	Isochlorogenic acid	$C_{25}H_{24}O_{12}$	515.1194	515.1197	1.348	17.03
60	Ethyl danshensu	$C_{11}H_{14}O_5$	225.0768	225.0756	-0.070	17.93
61	4-Coumaroylquinic acid	$C_{16}H_{18}O_8$	337.0928	337.0903	-1.494	18.15
62	3-p-Coumaroylquinic acid	$C_{16}H_{18}O_8$	337.0928	337.0908	-0.974	18.43
63	Aspidinol	$C_{12}H_{16}O_4$	225.1121	225.1108	-1.326	18.69
64	4-O-Feruloylquinic acid	$C_{17}H_{20}O_9$	367.1034	367.1015	-0.769	19.02
65	Hyperoside	$C_{21}H_{20}O_{12}$	463.0881	463.0858	-1.222	19.05
66	5-O-Feruloylquinic acid	$C_{17}H_{20}O_9$	367.10345	367.1012	-1.159	19.46
67	Serine	$C_3H_7NO_3$	106.04986	106.04776	-2.100	19.57
68	Acoramine C	$C_{15}H_{20}N_2O_6$	323.12485	323.12485	-0.743	19.80
69	p-Coumaric acid	$C_9H_8O_3$	163.04006	163.03798	-0.991	19.83
70	p-Hydroxyphenylacetic acid	$C_9H_8O_3$	165.05462	165.05356	-1.061	19.88
71	Cynaratriol	$C_{15}H_{22}O_5$	281.13944	281.13733	-1.020	19.99
72	Butylphthalide	$C_{12}H_{14}O_2$	191.10665	191.10532	-1.336	20.07
73	(E)-3-(2,4,5-Trimethoxyphenyl) acrylaldehyde	$C_{12}H_{14}O_4$	223.09648	223.09465	-1.835	20.13
74	Glucose	$C_6H_{12}O_6$	179.05611	179.0542	-0.815	20.39
75	Methyl caffeate	$C_{10}H_{10}O_4$	195.0651	195.0641	-1.005	20.44
76	5,7,4'-Trihydroxy-3',5'-dimethoxyflavone	$C_{17}H_{14}O_7$	329.0666	329.0648	-0.759	20.58

Compound	Metabolites	Formula	theoreticalm/z	m/z	MassError (ppm)	Retentiontime (min)
77	5-Coumaroylquinic acid	$C_{16}H_{18}O_8$	337.0928	337.0906	-1.154	20.62
78	Loliolide	$C_{11}H_{16}O_3$	197.1172	197.1163	-0.831	20.63
79	Tataroside	$C_{26}H_{44}O_{12}$	547.2759	547.2730	-1.833	20.77
80	Ferulic acid	$C_{10}H_{10}O_4$	193.0506	193.0486	-0.905	21.36
81	Kaempferol glucuronide	$C_{21}H_{18}O_{12}$	461.0725	461.0744	2.978	21.43
82	Isoorientin	$C_{21}H_{20}O_{11}$	447.0932	447.0904	-1.704	21.46
83	Kaempferol 3-O-glucoside	$C_{21}H_{20}O_{11}$	447.0932	447.0904	-1.708	21.46
84	Isoschaftoside	$C_{26}H_{28}O_{14}$	565.1551	565.1538	-1.372	21.53
85	Tyrosine	$C_9H_{11}NO_3$	182.0811	182.0800	-1.110	21.69
86	Eupafolin	$C_{16}H_{12}O_7$	315.0510	315.0503	0.421	21.74
87	Tataramide A	$C_{17}H_{17}NO_4$	298.1084	298.1042	-3.104	21.83
88	Ethyl caffeate	$C_{11}H_{12}O_4$	207.0662	207.064	-0.685	22.27
89	Vitexin	$C_{21}H_{20}O_{10}$	433.1129	433.110	-2.423	22.47
90	(S)-N-trans-Feruloyloctopamine	$C_{18}H_{19}NO_5$	328.1190	328.1170	-0.339	22.67
91	4,7,9,9'-Tetrahydroxy-3,3'-dimethoxy-8-O-4'-neolignan	$C_{20}H_{26}O_7$	377.1605	377.1590	-0.390	22.86
92	Luteolin 7-O-glucuronide	$C_{21}H_{18}O_{12}$	461.0725	461.0690	-2.362	23.43
93	4-Methoxycinnamic acid	$C_{10}H_{10}O_3$	179.0702	179.0693	-0.931	22.47
94	5,6-Dihydroxyflavone 7-O-glucuronide	$C_{21}H_{20}O_{10}$	431.0983	431.0957	-1.513	23.52
95	Cynaroside	$C_{21}H_{20}O_{11}$	447.0932	447.090	-1.488	23.65
96	Rosmarinic acid ethyl ester	$C_{20}H_{20}O_8$	387.1085	387.1075	0.066	23.62
97	Tatarinoids B	$C_{12}H_{16}O_5$	241.1070	241.1050	-1.960	23.63
98	Kaempferitrin	$C_{27}H_{30}O_{14}$	577.1562	577.1532	-1.922	23.81
99	Syringaldehyde	$C_9H_{10}O_4$	183.0651	183.0636	-1.555	23.91
100	Hyperoside	$C_{21}H_{20}O_{12}$	463.0881	463.0858	-1.222	23.93
101	Quercetin	$C_{15}H_{10}O_7$	303.0499	303.048	-1.829	23.98
102	Diosmin	$C_{28}H_{32}O_{15}$	607.1668	607.1637	-1.987	23.58

Compound	Metabolites	Formula	theoreticalm/z	m/z	MassError (ppm)	Retentiontime (min)
103	Uridine	$C_9H_{12}N_2O_6$	245.0768	245.0770	0.187	23.75
104	Salvianolic acid B	$C_{36}H_{30}O_{16}$	717.1461	717.1441	-0.841	24.05
105	Daidzein	$C_{15}H_{10}O_4$	253.0506	253.05494	-0.055	24.09
106	Isoquercitrin	$C_{21}H_{20}O_{12}$	463.0881	463.0856	-1.412	24.15
107	Rutin	$C_{27}H_{30}O_{16}$	609.1461	609.1429	-2.071	24.34
108	Leaf alcohol	$C_{10}H_{12}O_3$	181.0859	181.0842	-1.661	24.41
109	Isorosmarinic acid glycoside	$C_{24}H_{26}O_{13}$	521.1300	521.1267	-2.197	24.56
110	Oleraciamide	$C_{12}H_{13}NO_3$	220.0968	220.0955	-1.240	24.66
111	Acorus alcohol	$C_{12}H_{16}O_4$	225.1121	225.1108	-1.276	24.82
112	2,4,5-Trimethoxybenzoic acid	$C_{10}H_{12}O_5$	213.0757	213.0741	-1.560	24.86
113	Salvianolic acid A	$C_{26}H_{22}O_{10}$	493.1140	493.1117	-1.163	25.06
114	1-(2,4,5-Trimethoxyphenyl)propan-1,2-dione	$C_{12}H_{14}O_5$	239.091	239.0900	-1.330	25.32
115	Acoramone	$C_{12}H_{16}O_4$	225.1121	225.1108	-1.326	25.39
116	Rosmarinic acid glucoside methyl ester	$C_{25}H_{28}O_{13}$	535.1457	535.1433	-1.307	25.50
117	Baicalin	$C_{21}H_{18}O_{11}$	445.0776	445.0756	-0.918	25.56
118	3,5-Dicaffeoylquinic acid	$C_{25}H_{24}O_{12}$	515.1194	515.1158	-2.562	25.59
119	Hinokiresinol	$C_{20}H_{18}O_6$	355.1176	355.1163	-1.285	25.73
120	Apigenin 7-O-glucuronide	$C_{21}H_{18}O_{11}$	445.0776	445.0751	-0.918	25.78
121	Butylated hydroxyanisole	$C_{11}H_{16}O_2$	181.1223	181.1214	-0.896	25.86
122	Rosmarinic acid	$C_{18}H_{16}O_8$	359.0772	359.0752	-0.914	25.99
123	Grosheimol	$C_{15}H_{20}O_4$	263.1288	263.1273	-0.466	26.12
124	Isoacoramone	$C_{12}H_{16}O_4$	225.1121	225.1107	-1.386	26.14
125	Isorhamnetin 7-O-β-D-glucopyranoside	$C_{21}H_{20}O_{11}$	447.0932	447.0906	1.442	26.22
126	Kaempferol 3-O-rutinoside	$C_{27}H_{30}O_{15}$	593.1511	593.1478	-2.201	26.34
127	Quercitrin	$C_{21}H_{20}O_{11}$	447.0932	447.0906	-1.518	26.43
128	Quercetin	$C_{15}H_{10}O_7$	301.0353	301.0357	1.491	26.44

Compound	Metabolites	Formula	theoreticalm/z	m/z	MassError (ppm)	Retentiontime (min)
129	Kaempferol 7-O-neohesperidoside	$C_{27}H_{30}O_{15}$	593.1511	593.1481	-1.966	26.56
130	N-trans-Feruloyltyramine	$C_{18}H_{19}NO_4$	312.1241	312.1224	-0.625	26.63
131	Syringaresinol	$C_{22}H_{26}O_8$	417.1554	417.1572	2.866	26.91
132	Cyperol	$C_{15}H_{24}O$	221.1899	221.18887	-1.212	26.96
133	Threonine	$C_4H_9NO_3$	120.0655	120.0632	-2.300	27.02
134	Azelaic acid	$C_9H_{16}O_4$	187.0975	187.0956	1.075	27.19
135	Takakin-8-glucuronide	$C_{22}H_{20}O_{12}$	475.0881	475.0830	-4.062	27.19
136	Tricin 7-O-β-D-glucopyranoside	$C_{23}H_{24}O_{12}$	491.1194	491.1167	-1.642	27.19
137	(7R,8R)-Virolin2	$C_{22}H_{28}O_6$	389.1958	389.1932	-2.625	27.33
138	Diosmetin	$C_{16}H_{12}O_6$	299.0561	299.0543	-0.635	27.7
139	Homoplantaginin	$C_{22}H_{22}O_{11}$	461.1089	461.1064	-1.388	27.76
140	2,6-Dimethoxypyran-4-one	$C_7H_8O_4$	157.0495	157.0488	-0.645	27.81
141	Tatarinoids A	$C_{12}H_{16}O_5$	241.1070	241.1051	-1.950	27.87
142	Zedoarondiol	$C_{15}H_{22}O_3$	251.1641	251.1625	-1.631	28.12
143	3-Hydroxy-4',5,7-trimethoxyflavone	$C_{18}H_{16}O_6$	327.0874	327.0861	-0.195	28.41
144	Methyl rosmarinate	$C_{19}H_{18}O_8$	373.0928	373.0903	-1.464	29.06
145	Naringenin	$C_{15}H_{12}O_5$	271.0611	271.0596	-0.410	29.88
146	Luteolin	$C_{15}H_{10}O_6$	285.0404	285.0387	-0.574	30.17
147	Linarin	$C_{28}H_{32}O_{14}$	593.1864	593.1839	-2.582	30.18
148	Curcumenone epoxide	$C_{15}H_{18}O_3$	247.1328	247.1312	-1.641	30.28
149	Kaempferol	$C_{15}H_{10}O_6$	285.0404	287.0387	-0.664	30.39
150	Swertisin	$C_{22}H_{22}O_{10}$	447.1285	447.1257	-2.843	30.69
151	Betuletol	$C_{17}H_{14}O_6$	313.0717	313.0701	-0.475	30.83
152	Ligraminol C	$C_{23}H_{28}O_6$	401.1958	401.1998	3.995	32.20
153	Baicalein	$C_{15}H_{10}O_5$	269.0455	269.0439	-0.500	32.37
154	Genistein	$C_{15}H_{10}O_5$	269.0455	269.0441	-0.290	32.43

Compound	Metabolites	Formula	theoreticalm/z	m/z	MassError (ppm)	Retentiontime (min)
155	Apigenin	$C_{15}H_{10}O_5$	269.0455	269.0441	-0.290	32.43
156	Isomaltulose	$C_{12}H_{22}O_{11}$	341.1089	341.1087	0.862	32.59
157	Acoramine A	$C_{17}H_{13}NO_3$	280.0968	280.0951	-1.700	32.74
158	Kaempferide	$C_{16}H_{12}O_6$	299.0561	299.0543	-0.635	32.85
159	Acotatarone C	$C_{15}H_{22}O_2$	235.1692	235.1678	-1.436	32.93
160	Tricin	$C_{17}H_{14}O_7$	329.0666	329.0659	0.401	33.24
161	Isorhamnetin	$C_{16}H_{12}O_6$	299.0561	299.0543	-0.695	33.33
162	Hispidulin	$C_{16}H_{12}O_6$	299.0561	299.0542	-0.725	33.56
163	Chrysophanol	$C_{15}H_{10}O_4$	253.0506	253.0491	-0.375	34.14
164	α-Asarone	$C_{12}H_{16}O_3$	209.1172	209.1161	-1.101	34.84
165	Rhein	$C_{15}H_8O_6$	283.0248	283.0251	1.466	34.89
166	Endesmin	$C_{22}H_{26}O_6$	387.1802	387.1802	-3.125	34.94
167	Lysine	$C_6H_{14}N_2O_2$	147.1128	147.1160	3.256	35.29
168	α-Dehydrocalamene	$C_{15}H_{20}$	201.1637	201.1626	-1.107	35.29
169	β-Asarone	$C_{12}H_{16}O_3$	209.1172	209.1161	-1.121	35.42
170	Isolipidiol	$C_{15}H_{22}O_4$	265.1478	265.1436	-0.426	35.54
171	Eugenol	$C_{11}H_{14}O_2$	179.1066	179.1056	-0.976	35.85
172	γ-Asarone	$C_{12}H_{16}O_3$	209.1172	209.1160	-1.161	36.20
173	Hydnocarpin	$C_{25}H_{20}O_9$	465.1180	465.1154	-2.529	37.04
174	Stearic acid	$C_{18}H_{36}O_2$	283.2642	283.2615	-1.647	37.35
175	Acacetin	$C_{16}H_{12}O_5$	285.0757	285.0741	-1.650	37.36
176	Wogonin	$C_{16}H_{12}O_5$	285.0757	285.0740	-1.740	37.55
177	Ethyl palmitate	$C_{18}H_{36}O_2$	283.2642	283.2617	-1.407	37.76
178	Isoacorane diol	$C_{15}H_{26}O_2$	261.1825	261.1812	-1.447	39.31
179	2α,3α,24-Trihydroxyurs-12,20(30)-dien-28-oic acid	$C_{30}H_{46}O_5$	487.3418	487.3404	-1.351	41.21
180	Prunella saponin A	$C_{38}H_{62}O_8$	645.4371	645.4321	-3.965	41.65

Compound	Metabolites	Formula	theoreticalm/z	m/z	MassError (ppm)	Retentiontime (min)
181	2α,3α,24-Trihydroxyolean-12-en-28-oic acid	$C_{30}H_{48}O_5$	487.3428	487.3402	-1.561	41.66
182	2α,3β,24-Trihydroxyolean-12-en-28-oic acid	$C_{30}H_{48}O_5$	487.3428	487.3403	-1.441	42.21
183	2α,3α,24-Trihydroxyolean-11,13(18)-dien-28-oic acid	$C_{30}H_{46}O_5$	487.3418	487.3406	-1.191	43.00
184	2α,3α,23-Trihydroxyolean-12-en-28-oic acid	$C_{30}H_{48}O_5$	487.3428	487.3402	-1.591	43.21
185	Isopimaric acid	$C_{20}H_{30}O_2$	303.2318	303.2298	-2.027	43.36
186	2α,3α,19α-Trihydroxyurs-12-en-28-oic acid	$C_{30}H_{48}O_5$	487.3418	487.3403	-1.471	43.73
187	2α,3α-Dihydroxyurs-12,20(30)-dien-28-oic acid	$C_{30}H_{46}O_4$	471.3468	471.3458	-1.024	43.75
188	Calamusenone	$C_{15}H_{22}O$	219.1743	219.1731	-1.202	44.04
189	Calamusin A	$C_{15}H_{22}O$	219.1743	219.1731	-1.202	44.04
190	9-Hydroxy-10,12-octadecadienoic acid	$C_{18}H_{32}O_3$	295.2278	295.2262	-0.571	45.28
191	2α,3α,23-Trihydroxyurs-12-en-28-oic acid	$C_{30}H_{48}O_5$	487.3418	487.3401	-1.621	45.81
192	Maslinic acid	$C_{30}H_{48}O_4$	471.3479	471.3452	-1.676	46.62
193	Prunelloside A	$C_{35}H_{54}O_9$	617.3695	617.3661	-2.260	46.21
194	2α,3α-Dihydroxyolean-12-en-28-oic acid	$C_{30}H_{48}O_4$	471.3479	471.3451	-1.706	47.54
195	2α,3β-Dihydroxyolean-12-en-28-oic acid	$C_{30}H_{48}O_4$	471.3479	471.3451	-1.706	47.96
196	Valine	$C_5H_{11}NO_2$	118.0862	118.0857	-0.465	47.97
197	Corosolic acid	$C_{30}H_{48}O_4$	471.3479	471.3452	-1.676	48.16
198	3β,23-Dihydroxyurs-12-en-28-oic acid	$C_{30}H_{48}O_4$	473.3625	473.3624	-0.136	46.67
