# Supplementary material for: Prunella vulgaris Compound Improves Sleep Architecture and Attenuates Hippocampal Injury in Insomnia Mice
Source: Life (Basel). 2026 Jul 31;16(8):1268. doi: 10.3390/life16081268 (PMC13514870; doi:10.3390/life16081268)
Supplement: Supplementary file 1 [file life-16-01268-s001.zip › Table S2.pdf]

Table S2. Quantitative determination of three selected chemical markers in the PVC batch used for the animal experiments.

Chemical marker	Content (mg/g, mean ± SD)	RSD (%)
Salviaflaside	1.2412 ± 0.0253	2.04
Rosmarinic acid	3.4656 ± 0.0514	1.48
Linarin	4.3512 ± 0.0703	1.62

Note: Data are expressed as mean ± SD from six separately prepared sample solutions of a single PVC batch. Contents are expressed as mg/g of the final PVC granules. PVC, Prunella vulgaris compound; SD, standard deviation; RSD, relative standard deviation.
